# Verification of Acuros XB dose algorithm using 3D printed low‐density phantoms for clinical photon beams

**DOI:** 10.1002/acm2.12299

**Published:** 2018-03-25

**Authors:** Rodolfo Zavan, Philip McGeachy, Joseph Madamesila, Jose‐Eduardo Villarreal‐Barajas, Rao Khan

**Affiliations:** ^1^ Department of Medical Physics Tom Baker Cancer Center Calgary AB Canada; ^2^ Department of Radiation Oncology Washington University School of Medicine St. Louis MO USA

**Keywords:** 3D Printing, Acuros XB, dose calculation algorithm, experimental verification, radiochromic film

## Abstract

The transport‐based dose calculation algorithm Acuros XB (AXB) has been shown to accurately account for heterogeneities primarily through comparisons with Monte Carlo simulations. This study aims to provide additional experimental verification of AXB for clinically relevant flattened and unflattened beam energies in low density phantoms of the same material. Polystyrene slabs were created using a bench‐top 3D printer. Six slabs were printed at varying densities from 0.23 to 0.68 g/cm^3^, corresponding to different density humanoid tissues. The slabs were used to form different single and multilayer geometries. Dose was calculated with Eclipse™ AXB 11.0.31 for 6MV, 15MV flattened and 6FFF (flattening filter free) energies for field sizes of 2 × 2 and 5 × 5 cm^2^. EBT3 film was inserted into the phantoms, which were irradiated. Absolute dose profiles and 2D Gamma analyses were performed for 96 dose planes. For all single slab configurations and energies, absolute dose differences between the AXB calculation and film measurements remained <3% for both fields in the high‐dose region, however, larger disagreement was seen within the penumbra. For the multilayered phantom, percentage depth dose with AXB was within 5% of discrete film measurements. The Gamma index at 2%/2 mm averaged 98% in all combinations of fields, phantoms and photon energies. The transport‐based dose algorithm AXB is in good agreement with the experimental measurements for small field sizes using 6MV, 6FFF and 15MV beams adjacent to various low‐density heterogeneous media. This work provides preliminary experimental grounds to support the use of AXB for heterogeneous dose calculation purposes.

## 1 INTRODUCTION

Radiation therapy relies on accurate patient dose planning and delivery to ensure that the patient receives the prescribed dose. With the development of treatment planning systems, fast and accurate algorithms are available for dose calculation. The recently introduced volumetric dose calculation algorithm Acuros XB (AXB) (Varian Medical Systems, Palo Alto, CA, USA) deterministically solves the linear Boltzmann Transport Equation (LBTE).^1^, ^2^, ^3^ This new algorithm distinguishes itself from other methods of dose computation, such as the Analytical Anisotropic Algorithm (AAA)^4^, ^5^, ^6^ and the Collapsed Cone Convolution (CCC) algorithm, by directly solving the LBTE compared to the convolution and superposition methods employed by AAA and CCC to calculate dose. The LBTE describes the macroscopic behavior of the radiation as it interacts with matter, such as the dose deposition over a spatial resolution of roughly 1 mm or greater. AXB approaches the LBTE by discretizing the variables in space, angle and energy and iteratively solves for the fluence. Dose in a voxel can be calculated by using an energy‐dependent response function based on either dose‐to‐water or the material properties of the voxel. Similar to the Monte Carlo method, AXB is capable of reporting dose using this dose‐to‐medium option. Unlike convolution and superposition algorithms (e.g., AAA, CCC), where the heterogeneities within a patient are handled using density‐based corrections to the dose kernels calculated in water, AXB explicitly models physical interactions with matter using the mass density and material type for each voxel of the CT dataset.

Acuros XB has previously been investigated and validated with Monte Carlo simulations (VMC++, EGS4 etc) in heterogeneous geometries^2^, ^7^, ^8^ and compared with other dose calculation algorithms.^9^ Inhomogeneities have been simulated in varying complexity in terms of geometry, density, and material compositions for different field sizes and energies.^3^ In most of these cases Monte Carlo simulations, which is generally considered the gold standard in dose calculation, were used as the main reference for the algorithm validation. In general, AXB provides a fast and accurate alternative to Monte Carlo calculations for patient dose calculation. This has been demonstrated by good Gamma agreement (>86% pass rates for 3%/3 mm) for heterogeneous settings (normal lung, very low density lung, and bone) when compared with MC calculations, and as an improvement over AAA in terms of improved accuracy and reduced computation time for lung VMAT plans.

To date, only a handful of experimental investigations validating AXB in different geometric scenarios and clinical setups have been conducted.^1^, ^3^, ^10^, ^11^, ^12^, ^13^, ^14^ The majority of studies involved comparing AXB calculated dose results with commonly used clinical algorithms: AAA and CCC methods. Kan et al. assessed the dosimetric impact of AXB on intensity modulated radiotherapy (IMRT) and RapidArc™ for locally persistent nasopharyngeal carcinoma when recalculated from AAA.^15^, ^16^ In this study, measurements were obtained using thermoluminescent dosimeters (TLDs) and an ionization chamber at discrete points. Han et al. used the Radiological Physics Centre (RPC) head and neck (H&N)^17^ and thorax phantom^18^ to evaluate AXB for IMRT and volume modulated arc therapy (VMAT), using TLDs and GafChromic EBT2 film to obtain absolute point dose and planar dose measurements, respectively. Furthermore, the majority of studies were limited to only using 6MV beam energy. The present investigation looks to provide much needed data on experimental validation of AXB for a variety of clinically relevant beam energies, particularly in the case of lung VMAT and SBRT treatments, in a variety of 3D‐printed, low‐density geometries.

Published dosimetric comparisons have shown that the main advantage of AXB is accurate dose calculation in low‐density tissues.^2^, ^18^, ^19^, ^20^ Since AXB uses a density‐to‐material mapping table, it would be prudent to verify the algorithm experimentally in the range of both high‐ and low‐density tissues. To experimentally validate the algorithm in both density ranges, phantoms need to be fabricated with the same tissue equivalent materials. In this work, we have investigated only the low‐density range for clinical energies of interest in the small field sizes, due to inherent limitations of the 3'D printing fabrication process employed. That said, the investigation of small field size and low‐density tissue range for AXB validation is of clinical relevance as these conditions are akin to those of lung SBRT treatments, which has been a topic of interest with respect to the application of AXB.^21^, ^22^, ^23^, ^24^, ^25^

To this end, we have designed and developed phantoms of consistent material composition but with variable densities using a desktop 3D printer. In the context of inhomogeneities in treatment planning algorithms, dosimetric scalability (via methods such as equivalent path length) and their validation is of particular interest.

To our knowledge, there is no economical and commercial equivalent of variable density plastics available in the market. This work looks to supplement the Monte Carlo validation of the AXB algorithm performed thus far, with experimental evidence for a variety of clinically relevant photon energies, in small fields and low‐density phantoms. This work is also unique in the dosimetric applicability of cost‐effective 3D desktop printing in a cancer centre.

## 2 MATERIALS AND METHODS

### 2.A Experimental setup and geometry

Phantom slabs were created for this study using a 3D printer ORION Delta 3D (SeeMeCNC; Ligonier, IN, USA). ORION is a Rostock delta‐style printer consisting of three motorized controlled arms to provide full motion of fabrication along three orthogonal directions. The phantom slabs were fabricated with the purpose of varying the mass density while maintaining chemical composition, using only low atomic number elements common to human tissue. Polystyrene slabs of 10 × 10 × 2.4 cm^3^ were 3D printed, varying the printing parameters to roughly mimic the composition and density range of lung tissue.^26^ For all of 3D printed objects in this study, we restricted the infill pattern to the default grid pattern. For every slab, the quality of printing was verified with subsequent computed tomography (CT) scans acquired with 3 mm slice thicknesses. The mass density was determined from Hounsfield‐Unit‐to‐density calibration. The quality of each print was evaluated by reviewing CT images to ensure a uniform and artifact‐free object interior. The orientation of each test slab was labelled for reproducibility and alignment between the simulation and the irradiation.

**Figure 1:**
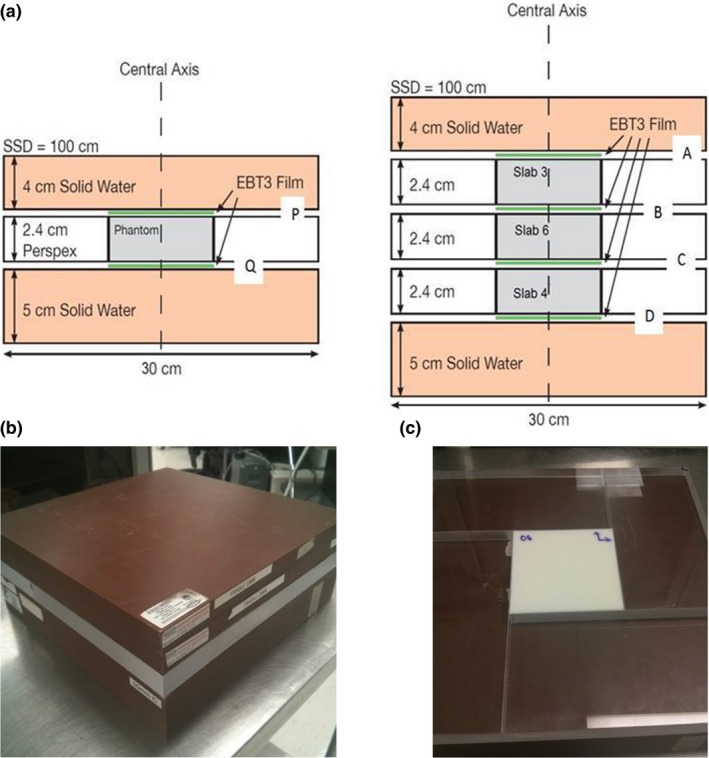
(a) Layout of the phantoms shown in axial view. Left: the single slab phantom geometry. Right: the multi slab phantom geometry. (b) Stack of solid water slabs used with 3D printed slabs. (c) 3D printed phantom with surrounding acrylic for centering and scattering purposes.

Each polystyrene slab was surrounded by 4 cm of Solid Water™ (Gammex‐RMI, Middleton, WI, USA) above and 5 cm below. Custom‐cut Perspex sheets were used to surround the slab laterally to minimize air gaps between layers. Figure 1 shows a schematic representation of the phantom setup for two geometries tested: single‐slab and multiple‐slabs. For the single‐slab, GafChromic EBT3 (Ashland Advanced Materials, Bridgewater, NJ, USA) films were placed above (depth P in Fig. 1) and below (depth Q in Fig. 1) the polystyrene slab. Variable density slabs were swapped to create 6 single‐slab phantom configurations. For the multi‐slab phantom, slabs 3, 6, and 4 (Table 1) were stacked with the films placed above, below and between each of the layers of polystyrene (depths A through D in Fig. 1). All seven phantoms were CT‐scanned with a 3 mm slice thickness.

**Table 1:** Mass density of the 3D‐printed polystyrene slabs. “SD” represents one standard deviation over the volume of the slab

	Mean density (g/cm^3^)	Mean HU ± SD
Slab 1	0.37	−645 ± 5
Slab 2	0.30	−713 ± 7
Slab 3	0.51	−502 ± 6
Slab 4	0.23	−785 ± 23
Slab 5	0.61	−391 ± 10
Slab 6	0.68	−330 ± 10

### 2.B. Dose calculation and measurements

The CT datasets were imported into Eclipse™ (Varian Medical System) treatment planning system, and dose calculations were performed for 6MV, 6FFF (6MV Flattening Filter Free) and 15MV beams from a Varian TrueBeam™ linear accelerator. The irradiations were planned for field sizes of 2 × 2 cm^2^ and 5 × 5 cm^2^ with source‐to‐surface distance (SSD) set to 100 cm. These smaller, clinically‐relevant field sized are typical of lung SBRT treatments, where we would expect more challenges for the dose calculation accuracy in low‐density media. A dose of 200 cGy was planned to a 4 cm depth (Fig. 1 depth P).

From the previous AXB studies,^1^, ^3^, ^10^, ^11^, ^12^, ^13^, ^14^ most of the institutions used a default dose grid of 2.5 mm for dose computation, therefore a calculation grid size of 2.5 mm was chosen for the AXB dose calculations with dose reported as dose‐to‐medium. AXB can also report dose‐to‐water; however, it was not pursued in our study.^27^ The decision of reporting dose‐to‐water or dose‐to‐medium has been a point of discussion in the past and justification of our choice in this study is provided in Discussion. No volume of the radiation field was allowed to travel through the lateral Perspex.

For a single slab phantom of low‐density 0.23 g/cm^3^ (Slab 4), dose was also computed with the commonly available AAA. Dose planes were extracted both at depths P and Q for the three energies and both field sizes.

All films and fabricated plastic slabs were aligned using external fiducial markings (BBs), placed on the phantom before CT simulation. The irradiations were done for all six single slab phantom setups and the multi‐slab setup, for two radiation fields and three beam energies with radiochromic films placed as shown in Fig. 1. Prior to each irradiation, the output of the linear accelerator was verified with an ionization chamber in a SolidWater™ phantom. The ionization chamber calibration is traceable to a primary standard at NRCC (National Research Council of Canada).

### 2.C Film dosimetry

GafChromic EBT3 film, which was designed for clinical dosimetry, was used in all of the studies. Small (4 × 4 cm^2^) EBT3 film calibration strips from the same batch were cut and marked for orientation. The films were reproducibly placed in a plastic template and scanned using an EPSON Expression 10000 XL flatbed scanner (US Epson, Long Beach, CA, USA). The films were scanned with transmission mode (positive film mode), 48 bits RGB (16 bits per channel color), 72 dpi resolution (0.35 mm/pixel), without any image correction. The GafChromic EBT3 film response is independent of energy for the range of MV photon energies investigated in this study.

The calibration film strips were placed at a depth of 5 cm in a 30 × 30 × 20 cm^3^ SolidWater™ phantom and irradiated with a 6MV linac beam for doses ranging from 0 to 8 Gy at 600 MU/min. The dose was subsequently measured in the same phantom at 5 cm depth with a calibrated ionization chamber traceable to the NRCC. The dose‐to‐water at 5 cm depth was determined from ionization measurements and using cross‐calibration factors related to absolute dosimetry using AAPM TG51 protocol guidelines.

All calibration film strips were scanned as previously described at the same location on the scanner, 24 ± 4 h after irradiation to ensure the optical density of the polymerized film has stabilized. Pre‐irradiation images were used to account for zero dose background intensity. The corrected images of all the strips were imported in the calibration module of DoseLab Pro version 6.50 (Mobius Medical Systems LP, Houston, TX, USA). EBT3 exhibits highest sensitivity (higher absorbance) at 636 nm; therefore, for dose evaluation, the maximum sensitivity is obtained using the red channel. According to the manufacturer, the red channel is recommended for dose evaluations up to 8 Gy. The resulting calibration plot was used for subsequent dose conversion for all irradiated films. More details about our film dosimetry protocol can be found in the literature.^28^

### 2.D Planar dosimetric comparison

For all experimental geometries, only one set of irradiations was performed. In total, 96 dose planes, 72 for the single analysis and 24 for the multi‐slab setup, calculated by Eclipse using AXB were exported and compared with the film measurements obtained from the GafChromic EBT3 films. We repeated irradiations for one configuration to verify the reproducibility. DoseLab Pro version 6.50 was used to perform film calibration and comparisons with calculated dose planes. Two‐dimensional local Gamma evaluation was performed for each film using 2% absolute dose and 2 mm distance‐to‐agreement criteria with a 10% dose cutoff threshold.^29^

## 3 RESULTS

Table 1 shows a summary of the physical properties of all printed polystyrene blocks. Based on the Hounsfield Unit (HU) of each voxel, density and material assignments are performed by AXB Version 11's material library. The variation in HU was found to be within 2‐4% of the mean value for all the slabs of various physical densities.

### 3.A Single slab phantom measurements

**Figure 2:**
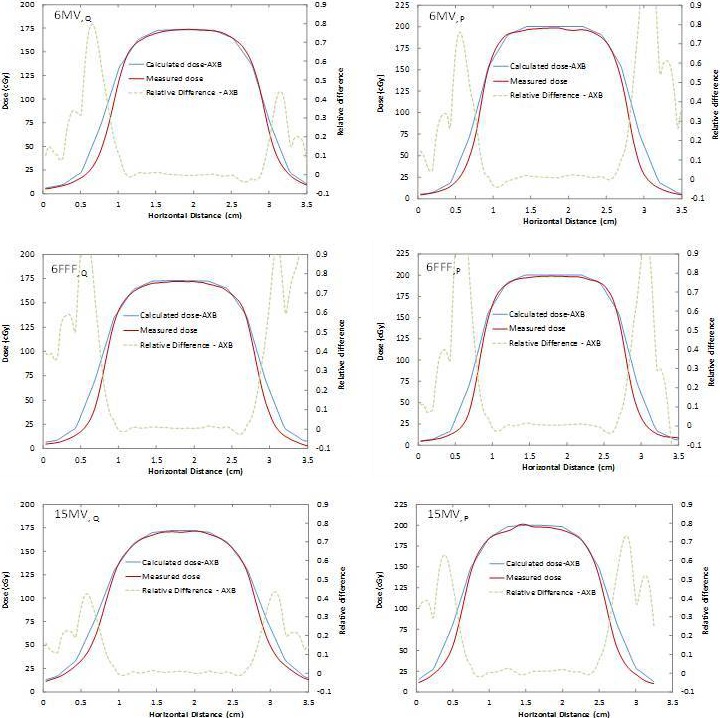
Absolute dosimetric profile comparisons for the 0.68 g/cm^3^ density slab for 6MV (top), 6FFF (middle), and 15MV (bottom) energies with 2 × 2 cm^2^ field size for depths Q (left) and P (right).

**Figure 3:**
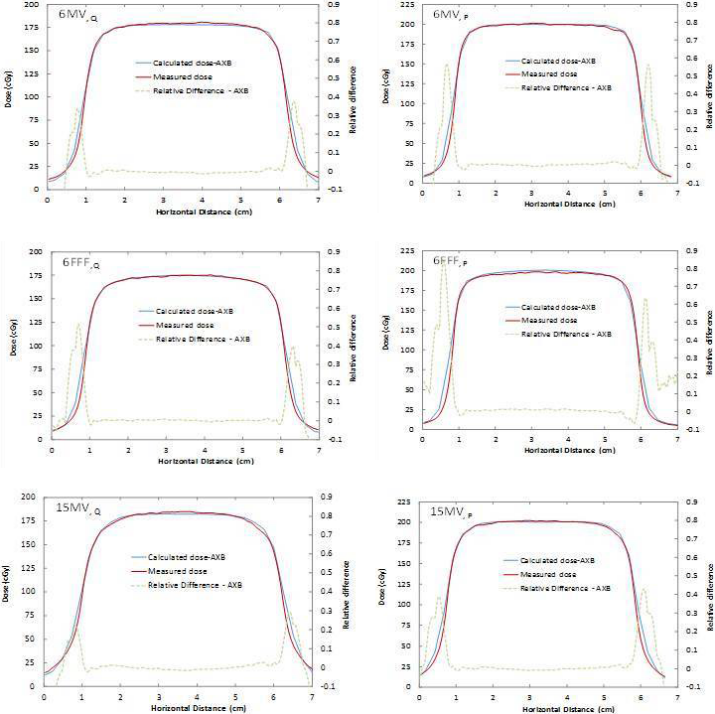
Absolute dosimetric profile comparisons for the 0.68 g/cm^3^ density slab for 6MV (top), 6FFF (middle), and 15MV (bottom) energies with 5 × 5 cm^2^ field size at depths Q (left) and P.

Absolute dose profiles were extracted in the cross‐plane direction from the films and compared with AXB calculated dose profiles for all configurations of slabs, energies and field sizes. Figures 2 and 3 show the profiles for the single slab phantom (density 0.68 g/cm^3^ [slab 6]) for field sizes of 2 × 2 and 5 × 5 cm^2^, respectively. The ratio of measured‐to‐calculated dose is also superimposed onto each profile. Dotted horizontal lines (±3% relative error) are drawn for guidance. All measurements and computations were found to be within 3% of each other, excluding the 90–10% penumbra regions.

**Figure 4:**
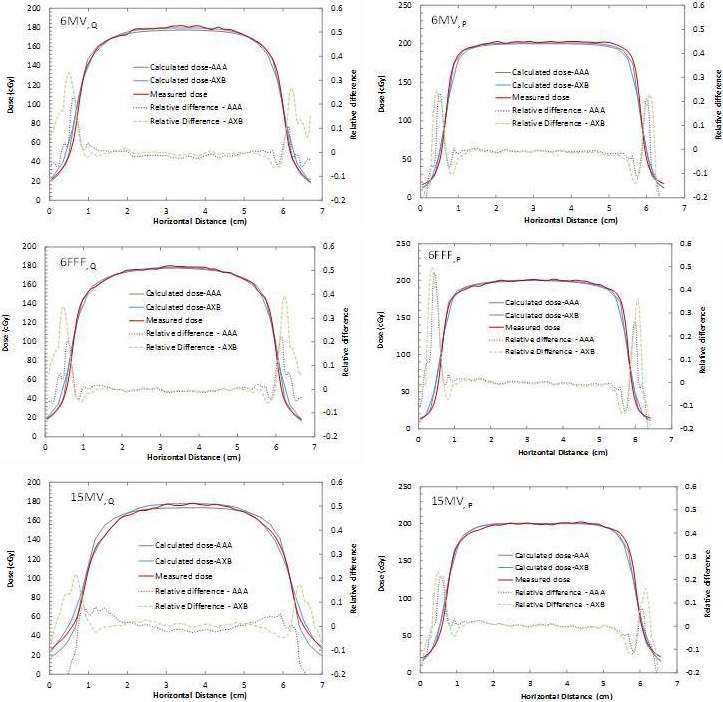
Absolute dosimetric profile comparisons (AXB, AAA, and Film measurements) for a slab of density 0.23 g/cm^3^ for 6MV (top), 6FFF (middle), and 15MV (bottom) energies with 5 × 5 cm^2^ field size.

Absolute dose profiles were extracted from the films at depth P and Q for Slab 4 (slab with lowest available density of 0.23 g/cm^3^) and compared with corresponding computed profiles from AAA and AXB for the three energies and the 5 × 5 cm^2^ field size. Figure 4 shows the absolute profiles and relative difference between computation and measurements. The results show that both algorithms were within 3% of the film measurements for all three energies in the high‐dose region.

For all planar doses, 2D Gamma analyses were performed. Table 2 shows failure rates for each absolute 2D Gamma analysis for 6MV, 6FFF and 15 MV beams and the stated 2%/2 mm criteria. All measured Gamma data showed pass rates ranging from 96.7% to 100% for all three beam energies and both field sizes at depths Q and P in the single slab phantoms.

**Table 2:** Absolute 2D Gamma failure rates, 2%/2 mm criteria at depths P and Q for the single‐layer configuration, and depths A, B, C, and D for the multi‐layer configuration

Density (g/cm^3^)/Slab #	Depth	6MV		6FFF		15MV	
		5 × 5	2 × 2	5 × 5	2 × 2	5 × 5	2 × 2
*Single layer phantom configuration*							
0.37/Slab 1	Q	0.2	0.0	1.0	0.0	0.3	0.2
	P	0.3	0.0	3.3	0.0	1.7	0.0
0.30/Slab 2	Q	0.4	0.0	1.2	0.0	1.2	0.1
	P	2.5	0.0	2.3	0.1	1.0	0.0
0.51/Slab 3	Q	0.5	0.0	0.3	0.0	0.6	0.0
	P	0.7	0.9	0.4	0.0	0.4	0.0
0.23/Slab 4	Q	0.2	0.0	0.2	0.0	0.9	0.1
	P	0.2	0.0	0.4	1.5	2.1	0.0
0.62/Slab 5	Q	0.5	0.0	0.4	0.0	0.2	0.0
	P	0.5	0.0	0.3	0.0	1.7	0.1
0.68/Slab 6	Q	0.3	0.0	0.3	0.0	0.2	0.0
	P	0.5	0.1	0.2	0.3	0.4	0.0
*Multilayer phantom configuration*							
	A	0.9	1.4	0.1	2.0	0.1	0.0
	B	0.1	4.2	1.5	0.4	0.0	0.0
	C	2.0	0.1	2.9	1.8	2.7	0.0
	D	0.9	2.3	0.6	0.0	0.7	1.7

### 3.B Multi‐slab phantom measurements

**Figure 5:**
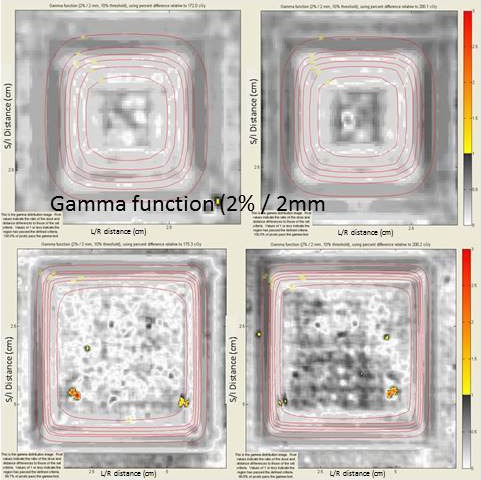
Planar gamma index for the 2 × 2 cm^2^ 15MV beam (top) and 5 × 5 cm^2^ 6FFF beam (bottom) at depths Q (left) and P (right) with a slab of density 0.68 g/cm^3^.

Reasonable profile agreement was observed at all depths in the multi‐slab phantom to within 3% of computation. Table 2 also summarizes the 2D Gamma analysis for the multi‐slab phantom geometry for all energies, field sizes, and depths (A through D). As a whole, all Gamma indices were able to achieve >95% pass rate with the majority of indices exceeding a high pass rate of ~98% for 2%/2 mm evaluation criterion for all energies and both field sizes in the multi‐slab heterogeneous phantom. Using discrete film measurements along the central axis of the beam, a comparison was made with the computation (Fig. 5). Error bars representing one standard deviation of the mean of the region of interest around the central pixel are given for each measurement.

## 4 DISCUSSION

AAPM task group report (TG‐65)^30^ on tissue heterogeneity management in radiation therapy enlists many challenges, most notably radiation transport through bone, air passages, cavities, and lung. With the exception of Monte Carlo simulation of radiation transport, modeling multiple scattered photons and high‐energy electrons remains difficult for most of the treatment planning algorithms used today, especially for high‐energy beams in low‐density media.^18^, ^31^ Introduction of the transport‐based solver Acuros XB has provided another venue to more accurately compute dose deposition in addition to Monte Carlo simulations. Adequately modeling primary beam attenuation, scattering of photons and transport of high‐energy electrons as they pass through media of different densities and compositions requires proper consideration of the physical properties of the medium and its influence on radiation. AXB accurately models the complex photon and electron transport in heterogeneous tissues by explicitly taking into account the material type and chemical composition.^4^, ^5^, ^6^

Validation of this dose algorithm in heterogeneous regions is an important task, and few studies have verified the algorithms through measurements. Benchmarking of such algorithms is often performed using Monte Carlo simulation with digital phantom geometries. The previously reported experimental methods have been either limited to point or relative measurements with different materials of variable compositions,^10^, ^11^, ^12^, ^13^, ^14^ thereby restricting their use for testing an algorithm which explicitly accounts for material compositions.

In the context of validating AXB, using phantoms of the same material with varying densities is of critical importance due to the algorithm's ability to model inhomogeneities using material composition. This work is the only example where custom‐built polystyrene slabs have been created, providing consistent chemical composition while allowing for variation in mass density. This was accomplished by printing custom slabs with relatively new 3D printing technology. Three‐dimensional printing is igniting interest in many different areas of radiotherapy such as the development of 3D printed electron bolus.^32^ We have shown that a simple, inexpensive, and desktop‐based printer can fabricate uniform and homogeneous phantom materials with minimal effort.^26^ However, one of the limitations of the presented technique is the inability to print and fabricate high‐density humanoid tissue objects. The challenge is posed by the availability of high‐density tissue equivalent plastic filaments. Though there are a few high‐density metal powders (iron, bronze, copper etc) and plastic mix filaments available, their radiological properties are quite different from bones and other high‐density tissues. The 3D fabrication methodology employed in our work with typical tissue equivalent plastics cannot exceed the density of the filament employed. Therefore, our current investigation was limited to low‐density object fabrication and their use for dose algorithm validation studies.

We experimentally tested the AXB in challenging geometries: both small fields and low‐density heterogeneous interfaces for 6MV, 6FFF and 15 MV clinical energies. The choice of energies in our study was dictated by the TrueBeam™ configuration available in our case. We chose GafChromic EBT3 films owing to their flexibility, and energy independence for MV dosimetry. Compared to ion chamber point dose measurements, the films provide higher spatial resolution and nondestructive, full dosimetric maps in a phantom. This was critical for the experimental validation performed in our study.

For the single slab phantom geometry (Fig. 2 and Table 2), excellent agreement was found between AXB and the GafChromic film measurements for 5 × 5 cm^2^ beams, with relative dose error below 3%. This agrees with Han et al. who observed AXB calculations matching discrete TLD measurements to within 5% using a RPC Head and Neck phantom,^17^ and Rana et al., who reported differences in up to 3%^10^ (only 6MV beams were used in both studies). For a small field of 2 × 2 cm^2^, the agreement also remained within 3% of dose measurements close to the center axis of the beam, deteriorating only in the penumbra region of the beam. Kan et al.^16^ investigated the differences in dose at distal interfaces using 6MV beams and AXB for 2 × 2 cm^2^ fields and obtained differences in up to 6% across media interfaces.^15^ AXB agreed with Monte Carlo simulated percent depth dose to within 2% in that study. Sato et al. also studied AXB in the build‐down region after lung‐water interfaces with a 4MV beam and 4 × 4 cm^2^ open fields, concluding the accuracy of AXB to be 3% when comparing with measurements and with AAA.^15^ This is consistent with our measurements at depth Q, with absolute dose agreement also within 3%. The 6FFF and 15MV results cannot be compared to the literature due to a lack of small‐field results for these beam energies in other studies. Bush et al. found differences of up to 3% near heterogeneous lung interfaces and up to 4.5% near air cavities.^7^ The data, however, were solely obtained using Monte Carlo simulations with BEAMnrc /DOSXYZnrc as a benchmark.

The major source of disagreement between film measurements and AXB calculations in our work arises in the penumbra regions in both field sizes for all energies. Since AXB models only the radiation transport through the patient, a major component of dosimetric penumbra depends on how the quality of the multisource model in Eclipse compares to the user's commissioning measurements, and the size of the radiation detector used in acquiring commissioning data. A beam model generates the fraction of primary and scatter components of a linac beam as a result of an iterative optimization process over a range of measured profiles at various depths. For a given energy, the quality of the multisource model is a compromise over all input profiles at field sizes from 3 × 3 cm^2^ to 40 × 40 cm^2^ at various depths in a water phantom during the beam modeling and configuration. In the case of AXB, source size is the only variable available to adjust the penumbra; the Beam Configuration workspace does not provide the flexibility to allow for manual tweaking. The secondary source distance in the modelling step can also have an impact on the size of the penumbra. A coarse CT slice thickness of 3 mm parallel to the beam direction could also result in voxel averaging for 3D printed low‐density objects, especially in the penumbra. Although strict alignment of printed slabs was observed between the simulation and measurements, slight variations from setup to setup could also result in disagreements, especially close to field edges.

We used two tools for analysis: (1) The absolute dose profiles along the cardinal axes and (2) planar dose comparison using 2D Gamma analysis. The profiles require absolute alignment between the measurement and computations. This method is affected by step‐size resolution, especially in the penumbra, and suffers when using the same evaluation criteria for the high‐dose regions and penumbra. Gamma analysis is somewhat forgiving in terms of alignment using distance to agreement along with the absolute doses.

The AAPM TG 53 report recommends a 3%/3 mm acceptance criterion between calculated and measured dose distributions for commissioning a treatment planning system.^33^ Two‐dimensional Gamma analysis with stricter criteria for open fields as a complement to dose profiles should be used as a viable tool for commissioning treatment planning systems. Therefore, 2D Gamma analysis was performed throughout the study for all fields and energies using more stringent Gamma criteria of 2%/2 mm for all single phantom measurements. Except for a few instances (all data ranged from 96.7% to 100% Gamma pass rates), our Gamma index analysis using the aforementioned criteria resulted in a ~98% pass rate of pixels. Han et al. also analyzed the Gamma index using Gamma criteria of 3%/3 mm and obtained agreement of >97% of pixels using film measurements.^14^ However, Fogliata et al. calculated an average agreement of 86% with 3%/3 mm adjacent to lung.^2^ In that study, 2D Gamma analysis for VMC++ simulated data was done adjacent to lung phantoms which is similar to the experimental setup used in our study. Gamma analysis is a difficult benchmark to compare with other studies due to lack of standard evaluation criteria.^29^, ^34^ Han et al.^17^ used different criteria when analyzing the 2D Gamma index such as 7%/4 mm, and considered the commonly used 3%/3 mm criteria too strict for their purposes.

In addition to delivery uncertainties of about 2–3% in our phantom irradiations, there are sources of uncertainty related to computational algorithms. In this study, AXB automatically assigned different material types to each pixel of the phantom; the material override feature was not considered. Therefore, for dose computation, density‐to‐material assignment for voxels of 3D printed slabs resulted in using low‐density lung, adipose tissue, muscle, and their combinations as the material of the voxels. The slight difference in chemical composition can produce a small disagreement. The auto‐assignment of materials represents the real clinical scenario—the way AXB is designed for automatic voxel segmentation for biological materials in patients. Comparison with dose‐to‐water reporting by AXB was avoided in our studies. It has been shown that the conversion of dose‐to‐medium to dose‐to‐water using stopping power ratios, as computed by Monte Carlo and AXB, may be substantially (up to 11%) different.^27^

**Figure 6:**
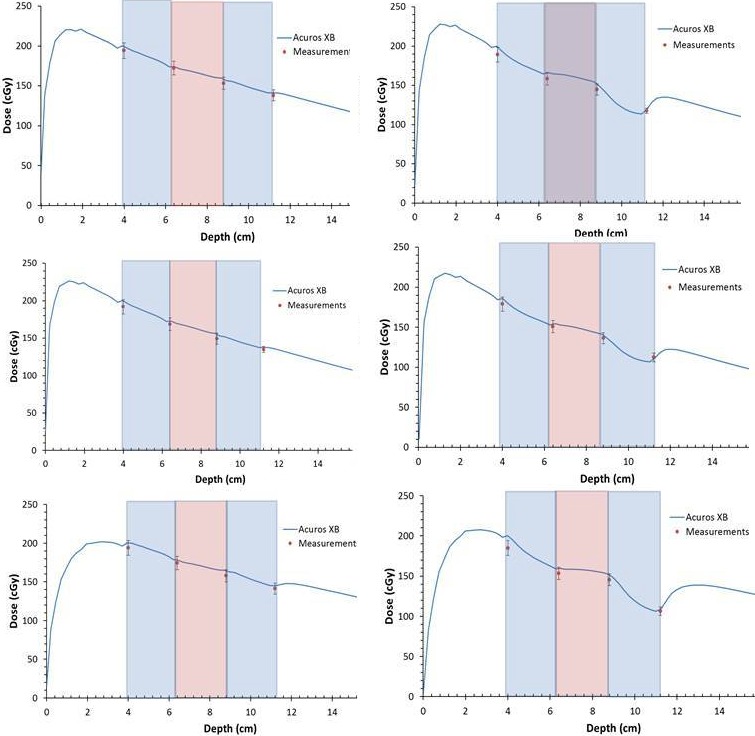
Central axis depth dose plots for the multi‐slab geometry. Energies shown are 6MV (top), 6FFF (middle), and 15MV (bottom) for field sizes 5 × 5 cm^2^ (left) and 2 × 2 cm^2^ (right). Media used from left to right: Solid Water, slab 3 (0.51 g/cm^2^), slab 6 (0.68 g/cm^2^), slab 4 (0.23 g/cm^2^), and Solid Water. Discrete film measurements are plotted. The error bars represent one standard deviation of the mean of an ROI of 3 × 3‐pixel width around the center. Color overlay of rectangular blocks approximately shows the various density slabs.

In the multi‐slab phantom configuration, dose profile agreement is achievable within 3% of measurements at all depths. Table 2 lists 2D planar Gamma analysis for all energies and the two field sizes; in all cases 97.1–100% of points pass the Gamma criteria. This is a strong indication of accurate modeling of radiation transport through multi‐density layers for the tested 6MV, 6FFF and 15 MV beams. Central axis discrete depth dose (Fig. 6) show that the achievable agreement is within 5%. This may be due to mixture voxels created at each interface in the phantom and the coarse spacing of the dose grid.^35^ The mixture voxels consisting of both air and plastic are created along all interfaces between these materials. The voxel material is an average of air to plastic contents available in a voxel. The measured dose is systematically lower due to the formation of an air gap for the shallow layer, resulting in a slight offset in the rest of the interfaces.

Several sources of uncertainty need to be considered when interpreting the film measurements. In this study, all measurements were performed using GafChromic ETB3 films commissioned with an inherent inaccuracy of 2%, which remains the main contributor to uncertainties in the measurements.

TrueBeam™ output was recorded prior to measurements and the variation was found to be <1% of the beam output. Uncertainties in setup were thoroughly analyzed for potential variations between CT simulation and actual radiation delivery. Dose planar measurements were done above and below 3D printed slabs, which resulted in various interfaces causing an uncertainty of about 2 mm due to the reproducibility of setup. This can cause a dose variation of about 2% at depths P and Q. Uncertainty due to SSD setup was determined to be <1 mm. The 3D printed blocks can vary by as much as ±0.5 mm in thickness between each block, and has the potential for creating a small air gap adjacent to the upper interface at depth P. Each layer of EBT3 film is approximately 0.25 mm thick which can add up to 1 mm for a multi‐slab phantom geometry. Table 3 provides a concise summary and description of various sources of uncertainty.

**Table 3:** Uncertainty analysis for experimental validation of AXB using 3D printed polystyrene slabs

Source of uncertainty	σ (%)	Description
EBT3 film dosimetry	2	Inherent dosimetric uncertainty due to film noise, background etc
Linac output variation	1	Daily output relative to reference measurements
Phantom setup reproducibility (±2 mm)	1	Obtained from repeated measurements of a given geometrical configuration
Source‐to‐surface distance (±1 mm)	1	Optical distance indicator uncertainty from 90–105 cm SSD
3D printed slabs (±0.5 mm)	0.5	Geometric reproducibility of 3D fabricated block
Air gap formation (1–2 mm)	1	Air gap formed between different layers of phantom
Dose to water vs. dose to film	0.5	For EBT film
Overall uncertainty	3	

Another source of uncertainty arises from overlapping dosimetric quantities—dose‐to‐water, dose‐to‐medium and dose‐to‐plastic in our experiments. The quantity measured with film is dose‐to‐water, which is obtained by converting optical density to dose. EBT3 film is known to be tissue equivalent in the Compton interaction range of energies and low Z_eff_ for the low‐energy component of MV photons.^18^, ^26^, ^28^ The treatment machine is calibrated in terms of dose‐to‐water according to the national and international dosimetry protocols. The differences between dose‐to‐water and dose to most soft tissues are clinically insignificant (within 2%).^31^, ^36^, ^37^ The overall uncertainty of all the setup and delivery, taken in quadrature (as in Table 3), is estimated to be below 3%.

Through this work, we have provided an experimental framework for validation of transport‐based dose calculation in single‐slab and multi‐slab low‐density geometries for common clinical beam energies. By developing custom phantoms using materials tailored to specific clinical needs, one can characterize the specific modeling capabilities of new dose calculation engines.

## 5 CONCLUSIONS

The advanced dose algorithm AXB was found to provide satisfactory agreement with experimental measurements using 6MV and 15MV flattened photon beams, as well as for unflattened 6FFF beams in low‐density heterogeneous media. This work provides an experimental evaluation of AXB algorithm for dose calculations in the challenging scenario of small fields irradiating low‐density regions, such as lung and adipose tissue. This provides added confidence in using this dose calculation algorithm in clinically relevant scenarios, such as the treatment of small lesions with relatively small field sizes in regions located at or in close proximity to soft tissue, low‐density interfaces, such as SBRT treatments for non‐small cell lung cancer.

## CONFLICT OF INTEREST

The authors declare no conflict of interest.
